# Inter‐ and intra‐observer agreement of 2D and 3D transperineal ultrasound of pelvic floor hiatal measurements in the second stage of labor

**DOI:** 10.1111/aogs.70270

**Published:** 2026-06-16

**Authors:** Mariya Kovalenko, Giulia Zamagni, Ingrid Volløyhaug, Claudia Montero Carreras, Sana Usman, Arwa Hanidu, Tamara Stampalija, Lorenzo Monasta, Kjell Salvesen, Christoph Lees

**Affiliations:** ^1^ Department of Digestion, Metabolism and Reproduction Imperial College London London UK; ^2^ Centre for Fetal Care Queen Charlotte's and Chelsea Hospital, Imperial College Healthcare NHS Trust London UK; ^3^ Clinical Epidemiology and Public Health Research Unit Institute for Maternal and Child Health – IRCCS “Burlo Garofolo” Trieste Italy; ^4^ Department of Obstetrics and Gynecology St. Olavs Hospital Trondheim Norway; ^5^ Department of Clinical and Molecular Medicine Norwegian University of Technology and Science Trondheim Norway; ^6^ Department of Obstetrics, Gynecology, and Reproductive Medicine Institut Universitari Dexeus Barcelona Spain; ^7^ Department of Radiological Sciences, College of Health and Rehabilitation Sciences Princess Nourah Bint Abdulrahman University Riyadh Saudi Arabia; ^8^ Unit of Fetal Medicine and Prenatal Diagnosis Institute for Maternal and Child Health IRCCS “Burlo Garofolo” Trieste Italy; ^9^ Department of Medicine, Surgery and Health Sciences University of Trieste Trieste Italy; ^10^ Department of Development & Regeneration KU Leuven, Oude Markt 13 Leuven Belgium

**Keywords:** image scoring, imaging, intrapartum, levator ani muscle

## Abstract

**Introduction:**

Transperineal ultrasound has emerged as a reliable measure to monitor labor progress non‐invasively. This sonographic method enables fetal head descent to be measured by determining the relationship of the fetal head to the maternal symphysis pubis and the fetal head in reference to the perineum. As the pelvic floor plays an important role in childbirth, measuring the levator hiatal dimensions may provide additional understanding of the mechanisms, particularly in the second stage of labor.

**Material and Methods:**

The objective was to assess both inter‐ and intra‐observer agreement in measuring the levator hiatal dimensions at rest during the passive second stage of labor using two‐ and three‐dimensional (2D, 3D) transperineal ultrasound. Women were prospectively recruited for ultrasound assessment in the second stage of labor. The study population consisted of nulliparous women at term (37 + 0–42 + 0 weeks' gestation) with a live singleton pregnancy. Study participants eligible for recruitment were approached in active labor and consented to participate in the “SONO‐BIRTH” study. Transabdominal and transperineal ultrasound were performed following clinical assessments in labor. The 3D pelvic floor volumes were uploaded into the software to calculate the levator hiatal area (LHa), transverse hiatal diameter (TD), and the anteroposterior hiatal distance (APD). The quality of visualization from the 3D volumes of the symphysis pubis (SP), puborectalis (PR), and levator hiatus (LH) was scored between 0 and 2 (SP, PR) and 0–4 (LH) and was assessed by two independent observers.

**Results:**

Of 95 participants consecutively recruited, 75 had ultrasound volumes available for assessment. The inter‐observer agreement for APD in 3D was slightly better compared with APD in 2D measurements, the mean difference being −0.22 cm (intraclass correlation coefficient = 0.85; *p* < 0.001) and − 0.35 cm (intraclass correlation coefficient = 0.79; p < 0.001), respectively. Intra‐observer agreement for APD in 2D demonstrated a mean difference of −0.03 cm (intraclass correlation coefficient = 0.91; p < 0.001). Image quality of 3D ultrasound volumes gave a Kappa score of 0.40 for SP and a Kappa score of 0.72 and 0.77, respectively, for PR and LH.

**Conclusions:**

Assessment of the levator hiatal dimensions in the second stage of labor using 2D and 3D ultrasound demonstrates good inter‐ and intra‐observer agreement. Agreement between two operators for quality of imaging of the pelvic floor structures obtained from 3D imaging was lowest for the symphysis pubis and highest for the levator hiatus.

## Graphical abstract

Assessment of the levator hiatal dimensions in the second stage of labor using transperineal ultrasound is feasible with good inter‐ and intra‐observer agreement. Intrapartum ultrasound can improve our understanding of variation in pelvic floor anatomy during childbirth.

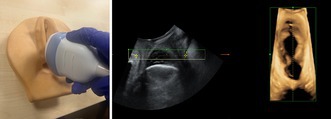

**Abbreviations**
2D: two‐dimensional
3D: three‐dimensional
APD: anteroposterior diameter
ICC: intraclass correlation coefficient
LAM: levator ani muscle
LH: levator hiatus
LHa: levator hiatal area
PR: puborectalis
SP: symphysis pubis
TD: transverse distance
TPUS: transperineal ultrasound

**Key message**

Performing transperineal ultrasound in the second stage of labor to obtain levator hiatal dimensions is feasible and results in good inter‐ and intra‐observer agreement.

## 1 INTRODUCTION

The pelvic floor is composed of an intricate soft tissue complex comprising the levator ani muscle (LAM), the vaginal wall, connective tissue, fascia, and ligaments, which collectively provide support to the pelvic organs.^1^ Pregnancy, labor, and vaginal birth can impact on the anatomy of the pelvic floor, particularly the LAM, through a combination of hormonal and mechanical factors.^2^

Transperineal ultrasound (TPUS) has enhanced our knowledge of the relationship between labor and pelvic floor disorders,^3^ allowing accurate assessment of the pelvic floor anatomy before and during labor offering the potential for predicting the mode of delivery^4^, ^5^, ^6^ and the risk of childbirth‐related perineal trauma.^7^, ^8^, ^9^ While the effectiveness of TPUS in evaluating the pelvic floor during pregnancy and postpartum is well‐established, its application during the intrapartum period remains underexplored. This gap is particularly notable during the late stages of labor when the most significant stretching of the pelvic floor muscles occurs. Computational models have quantified this phenomenon, showing that the LAM stretches approximately 2.5 to 3.26 times its original length to accommodate the fetal head.^10^, ^11^, ^12^

For intrapartum TPUS to be effectively integrated into clinical practice, it is desirable to establish the reproducibility of its technique, considering that image quality and assessment of pelvic floor landmarks may affect the overall hiatal measurements. Similar work has been done in evaluating inter‐ and intra‐observer agreement in measuring cervical dilatation^13^ across stages of labor and inter‐observer agreement for head descent measured by the angle of progression.^14^

A key challenge in intrapartum TPUS is the presence of the fetal head in the vaginal canal,^15^ obscuring local soft tissue structures. In the second stage of labor, evaluation of the fetal head station and fetal head position has been well studied.^16^, ^17^, ^18^ However, three‐dimensional (3D) ultrasound, particularly of the pelvic floor at the advanced labor stage, has not been extensively explored. Therefore, this study aimed to evaluate the inter‐ and intra‐observer agreement in 2D and 3D TPUS measurements of the pelvic floor during the second stage of labor, and to develop a grading classification for the quality of the 3D rendered images of the pelvic floor.

## 2 MATERIAL AND METHODS

Nulliparous women with maternal age 18 to 45 years were recruited to a prospective single‐centre cohort study from 2023 to 2025 (“SONO‐BIRTH” study). Participants were women with a term, cephalic, singleton pregnancy between 37 and 42 weeks of gestation in the late active first stage (≥ 7 cm) of labor who were approached on the Delivery Suite to participate. The exclusion criteria included life‐threatening maternal or fetal compromise requiring immediate medical intervention and/or delivery, as well as the inability to provide full consent for the study. Once consent was obtained, women underwent digital vaginal examinations as part of standard labor care. Once second stage of labor was confirmed by the clinical care team, transabdominal and transperineal ultrasound was performed prior to the onset of maternal pushing and in between uterine contractions. Ultrasound examinations were performed and recorded using portable ultrasound equipment (Voluson™ SWIFT, GE Healthcare), with a curved real‐time four‐dimensional (4D) and a 2‐5 MHz wide‐band three‐dimensional (3D) convex ultra‐light volume probe. The research team was not involved in the intrapartum clinical decision‐making and participants were blinded to the intrapartum ultrasound findings. There was no formal power calculation.

**FIGURE 1:**
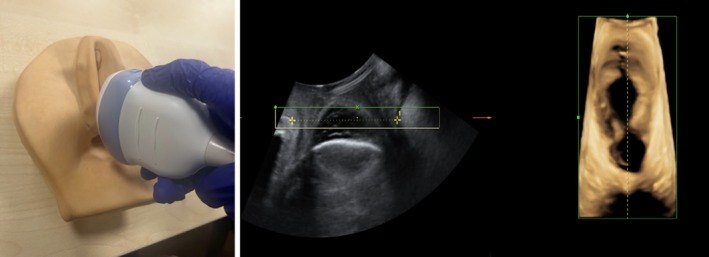
Transperineal 3D ultrasound in the midsagittal plane with corresponding 2D and 3D render image at the plane of minimal hiatal dimension.

Transperineal ultrasound performed in the midsagittal plane was used to obtain a 3D volume of the pelvic floor at the plane of minimal hiatal dimension (Figure 1). Participants were examined in a supine position with knees and hips flexed, at rest, between uterine contractions, with regional anesthesia dependent on maternal preference. The transducer was positioned between the labia majora at the posterior fourchette. First, 2D images were captured in the transverse and sagittal planes on cine‐loop videos to measure the head‐perineum distance (HPD) and angle of progression (AoP), respectively, according to the International Society of Ultrasound in Obstetrics and Gynecology (ISUOG) guidelines for intrapartum ultrasound.^19^ In the midsagittal plane, 3D static volumes were recorded at an acquisition angle width of 90°, with each automatic image acquisition lasting approximately 3 seconds. During participant recruitment, a minimum of two ultrasound volumes were obtained in the second stage of labor. Where the volume was unclear, the 3D probe was re‐positioned and further ultrasound volumes were acquired until the researcher was satisfied that the most optimum volume for the participant was captured. However, the final dataset included measurements from the technically best ultrasound volume.

### 2.1 Part 1: Inter‐ and intra‐observer agreement and agreement for 2D and 3D hiatal dimensions

**FIGURE 2:**
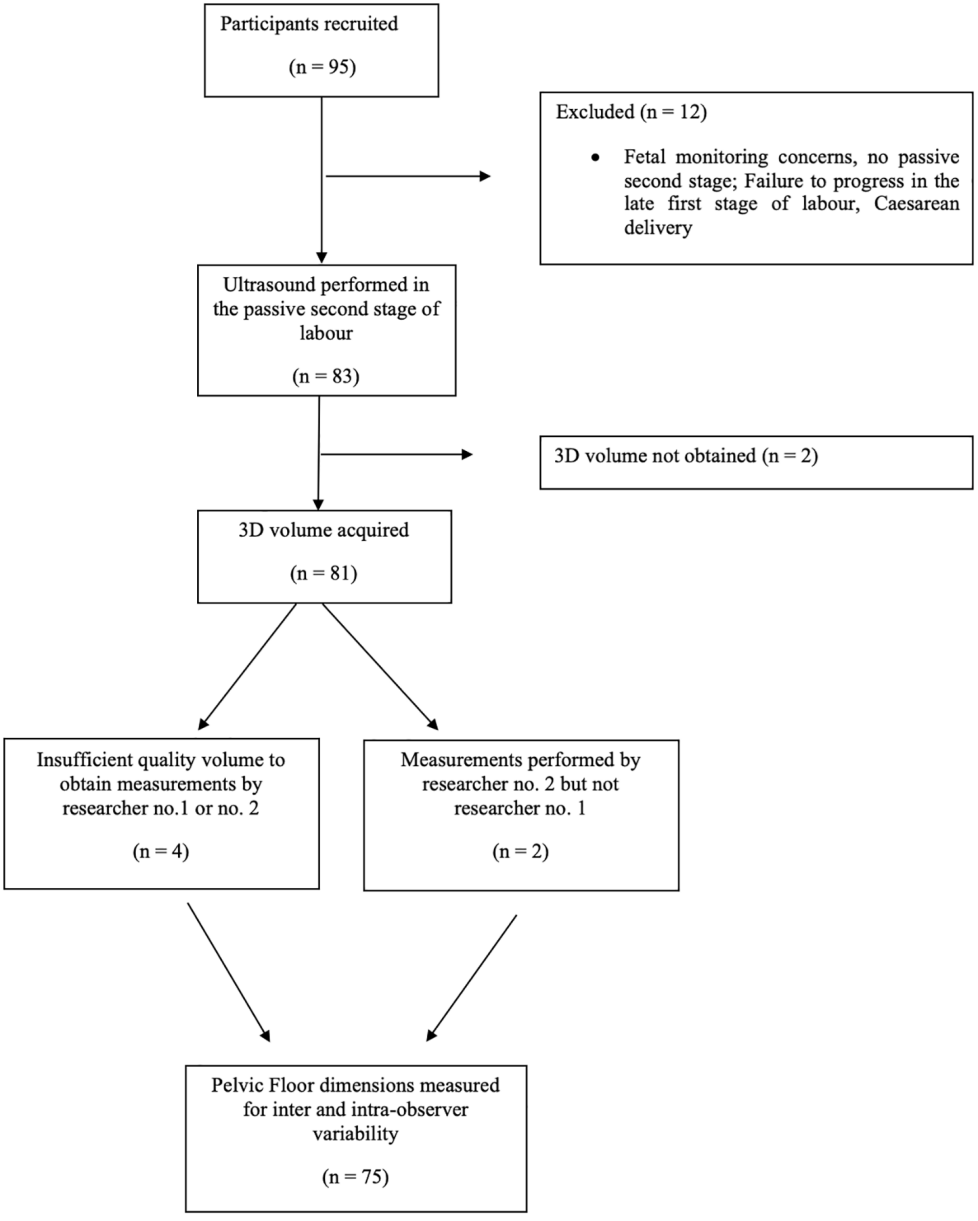
Flow chart depicting the study cohort.

Analysis of 2D images, cine‐loop videos, and 3D ultrasound volumes was conducted remotely from the labor room. The cineloops were reviewed by MK and frozen at the frame that demonstrated clear anatomical landmarks allowing for the optimal measurement. Ultrasound volumes were analyzed offline using 4D View version 18 software (GE Healthcare). Two independent researchers (MK and IV) participated in separately reviewing the 3D intrapartum volumes for inter‐observer agreement in measurements of the levator hiatal area (cm^2^), transverse hiatal distance (cm), and the anteroposterior hiatal diameter (cm). All investigators were clinical obstetricians and had extensive experience in undertaking ultrasound measurements. The levator hiatal dimensions were defined and measured according to the method described by Dietz et al.^20^ The anteroposterior diameter (APD) was assessed in the 2D image (midsagittal plane of the sectional planes) and in the rendered 3D volume of approximately 1 cm thickness,^21^ refer to Figure 2. Multiplanar reconstruction was used to correct for potential malalignment. While the 3D image acquisition was in the midsagittal plane, the measurement was made from a multiplanar reconstruction from the same plane that was taken from the 2D image. The transverse distance (TD) and levator hiatal area (LHa) were identified and measured exclusively in the axial plane using 3D TPUS. The standard 2D APD was compared with the APD measured from the 3D volume. The researchers were blinded to one another's measurements. One researcher (MK) repeated measurements after a three‐month interval for assessment of intra‐observer agreement. Pseudoanonymized data were entered into an Excel spreadsheet prospectively at the time of ultrasound; participants were identified by study number only. Bland–Altman analysis and intraclass correlation coefficient (ICC) were performed to evaluate agreement between measurements of the pelvic floor. ICC values were interpreted based on established criteria, where values below 0.50 indicate poor reliability, 0.50–0.75 moderate reliability, 0.75–0.90 good reliability, and values above 0.90 excellent reliability.

### 2.2 Part 2: Quality assessment score for the anatomical structures identified from 3D hiatal volumes

A scoring system was developed by the authors to assess the image quality of the 3D volumes, using a scale ranging from zero to eight. This scale subjectively evaluated the visibility and clarity of key anatomical landmarks: the symphysis pubis (SP), the puborectalis muscle (PR), and the complete outline of the levator muscle (LH) (Table 1). Two observers (MK and CM) blindly scored each 3D volume render at the plane of the minimal hiatal dimensions. The observers scored the SP, PR, and LH separately, and a total score was generated. Comparison was made between the scores of the two observers.

**TABLE 1:** Grade scoring system for 3D volume image quality.

Anatomical landmarks	3D volume recorded	Points
Distal echo of symphysis pubis	Clearly seen	2
	Partially seen^a^	1
	Not seen	0
Puborectalis muscle	Clearly seen	2
	Partially seen^a^	1
	Not seen	0
Levator hiatal outline	All 4 borders seen	4
	3 out of 4 borders seen	3
	2 out of 4 borders seen^a^	2
	1 out of 4 borders seen	1
	No borders seen	0

^a^ Minimal points required to achieve the total score of four necessary to reliably measure the dimensions of the levator hiatus.

Higher scores indicated better image quality as all landmarks were clearly visible, with a minimum total score of four required to reliably measure the dimensions of the levator hiatus.

## 3 RESULTS

Ninety‐five participants were consecutively recruited, of whom 12 did not have an ultrasound assessment due to fetal distress limiting time for an ultrasound assessment or the participant did not reach the second stage of labor following initial recruitment during the late first stage of labor. From 83 ultrasound assessments performed during the second stage of labor, in 2 cases, a 3D pelvic floor volume was not obtained. Eighty‐one pelvic floor ultrasound volumes were acquired and subsequently analyzed by two independent researchers. In four cases, a 3D volume was obtained but was of unsatisfactory quality to be measured by either researcher, and in a further two cases, the measurements were performed by the second researcher but not the first. Consequently, 75 pelvic floor ultrasound volumes were measured for inter‐ and intra‐observer agreement (Figure 2).

Demographic characteristics of the study participants included a mean age of 32 ± 4.8 years, a mean gestational age of 40 weeks ±1 week +3 days, and a mean body mass index (BMI) of 24.5 ± 4.3 kg/m^2^. In our dataset, the mean APD in 2D was 7.05 cm (SD = 1.05) and in 3D 7.07 cm (SD = 1.02). The mean LHa in 3D was 20.40 cm^2^ (SD = 6.00), and the TD was 3.98 cm (SD = 0.55). Further information on the levator hiatal dimensions is summarized in Figures S1 and S2.

### 3.1 Part 1: Inter‐ and intra‐observer agreement and agreement for 2D and 3D hiatal dimensions

**FIGURE 3:**
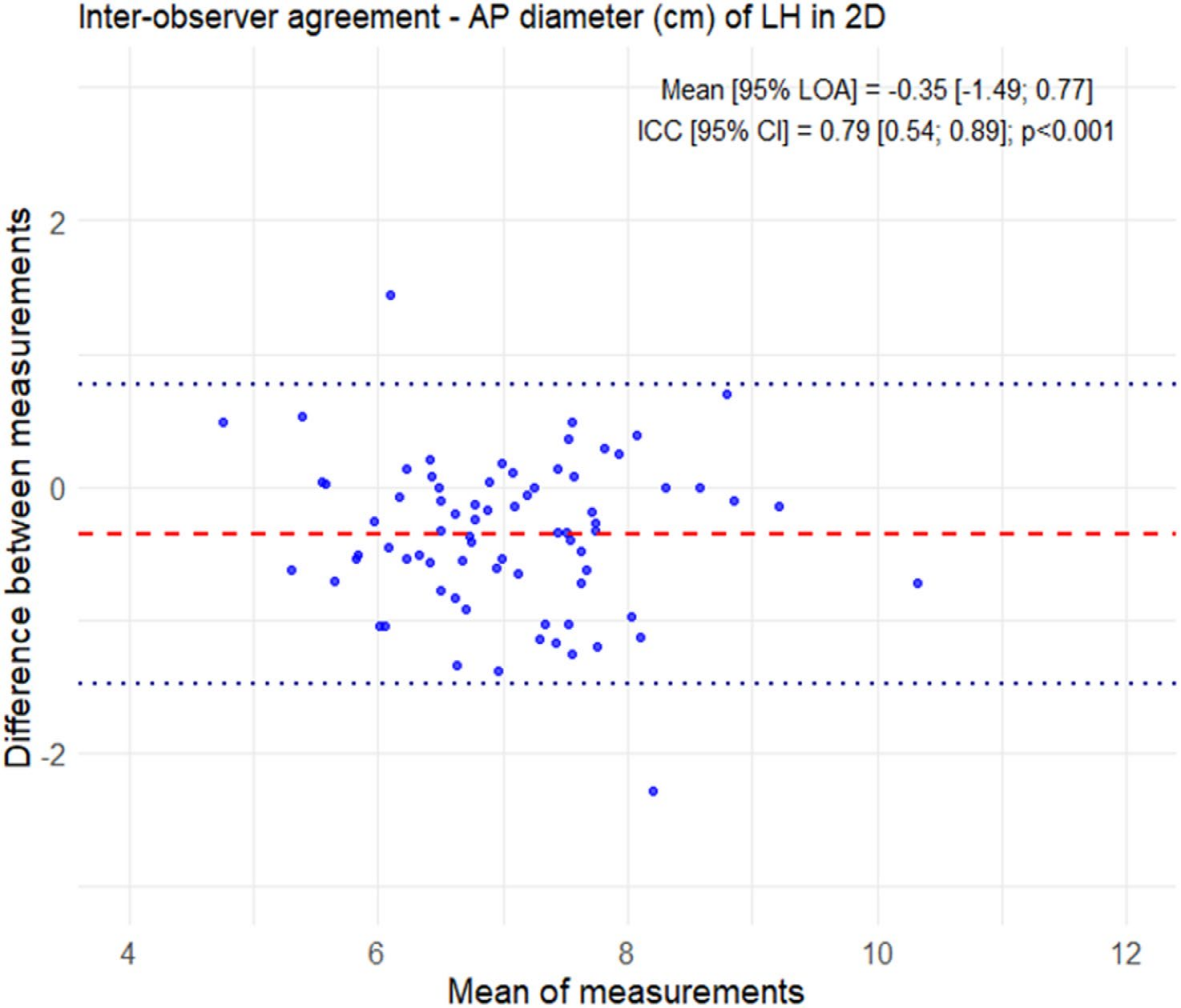
Bland–Altman plot with mean difference of the inter‐observer agreement of the anteroposterior (AP) diameter of the levator hiatus (LH) area during two‐dimensional (2D) imaging.

**FIGURE 4:**
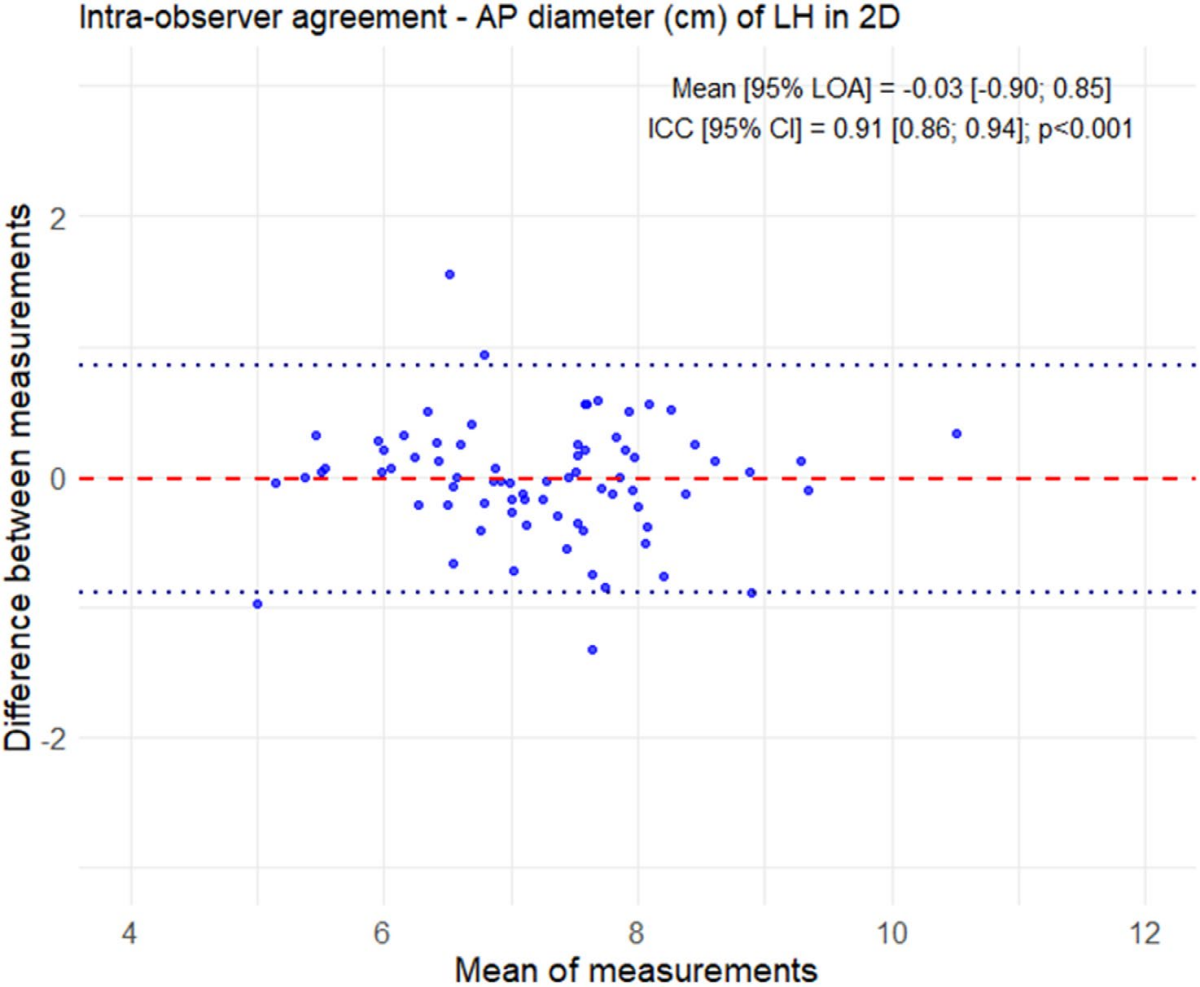
Bland–Altman plot with mean difference of the intra‐observer agreement of the anteroposterior (AP) diameter of the levator hiatus (LH) area during two‐dimensional (2D) imaging.

For inter‐observer agreement, APD in 2D and 3D measurements had a mean difference of −0.35 cm (ICC = 0.79; *p* < 0.001) and a mean difference of −0.22 cm (ICC = 0.85; *p* < 0.001), respectively. The intra‐observer agreement in APD in 2D and 3D demonstrated a mean difference of −0.03 cm (ICC = 0.91; *p* < 0.001) and a mean difference of −0.01 cm (ICC = 0.94; *p* < 0.001), respectively. Bland–Altman plots for inter‐ and intra‐observer agreement for APD in 2D are displayed in (Figures 3 and 4). Additional figures are found in Supporting Information (Figures S3–S5). Bland–Altman plots did not indicate evidence of proportional bias or heteroscedasticity.

**FIGURE 5:**
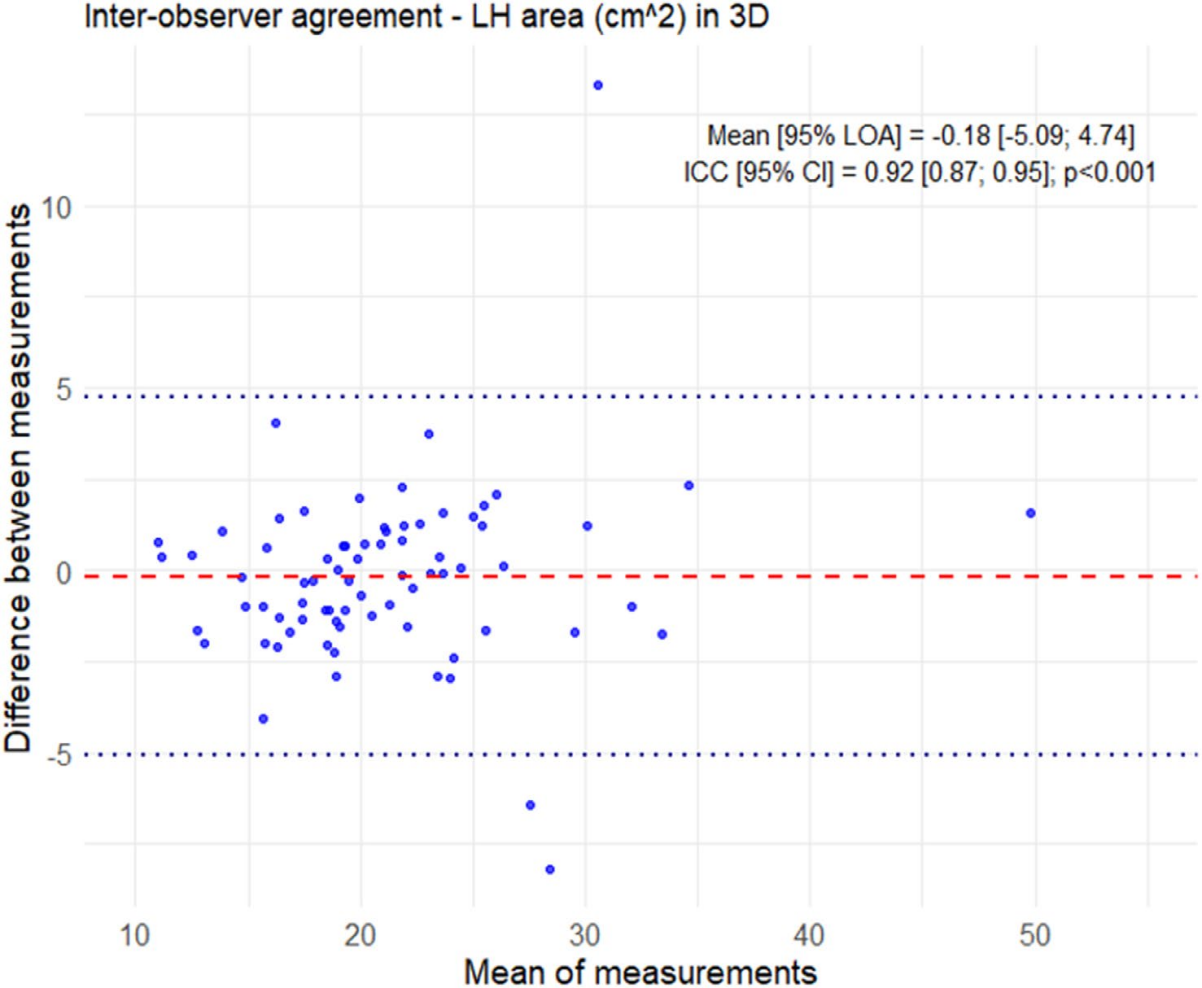
Bland–Altman plot with mean difference of the inter‐observer agreement in the levator hiatal (LH) area during three‐dimensional (3D) imaging.

The LHa mean difference of −0.18 cm (ICC = 0.92; *p* < 0.001) for inter‐observer agreement (Figure 5) and for intra‐observer agreement, the mean difference being −0.21 cm (ICC = 0.98; *p* < 0.001).

In the TD, a mean difference was observed of −0.07 cm (ICC = 0.82; p < 0.001) for inter‐observer agreement, and for intra‐observer agreement, the mean difference was −0.01 cm (ICC = 0.87; p < 0.001). A summary table of the results is shown in Table 2.

**TABLE 2:** Bland–Altman plot data of the differences between inter‐ and intra‐observer agreement in anteroposterior diameter (APD) measured in 2D and 3D ultrasonography, levator hiatal area (LHa), and levator transverse distance (TD): Mean difference; 95% limits of agreement (LOA); intraclass correlation coefficient (ICC).

Levator hiatal dimension	Inter‐observer agreement mean diff. (95% LOA)	ICC (95% CI)	*p* Value	Intra‐observer agreement mean diff. (95% LOA)	ICC (95% CI)	*p* Value
APD (cm) in 2D	−0.35 (−1.49; 0.77)	0.79 (0.54; 0.89)	< 0.001	−0.03 (−0.90; 0.85)	0.91 (0.86; 0.94)	< 0.001
APD (cm) in 3D	−0.22 (−1.23; 0.79)	0.85 (0.73; 0.91)	< 0.001	−0.01 (−0.67; 0.70)	0.94 (0.91; 0.96)	< 0.001
LHa (cm^2^) in 3D	−0.18 (−5.09; 4.74)	0.92 (0.87; 0.95)	< 0.001	−0.21 (−2.48; 2.89)	0.98 (0.96; 0.99)	< 0.001
TD (cm) in 3D	−0.07 (−0.74; 0.60)	0.82 (0.72; 0.88)	< 0.001	−0.01 (−0.52; 0.54)	0.87 (0.81; 0.92)	< 0.001

**FIGURE 6:**
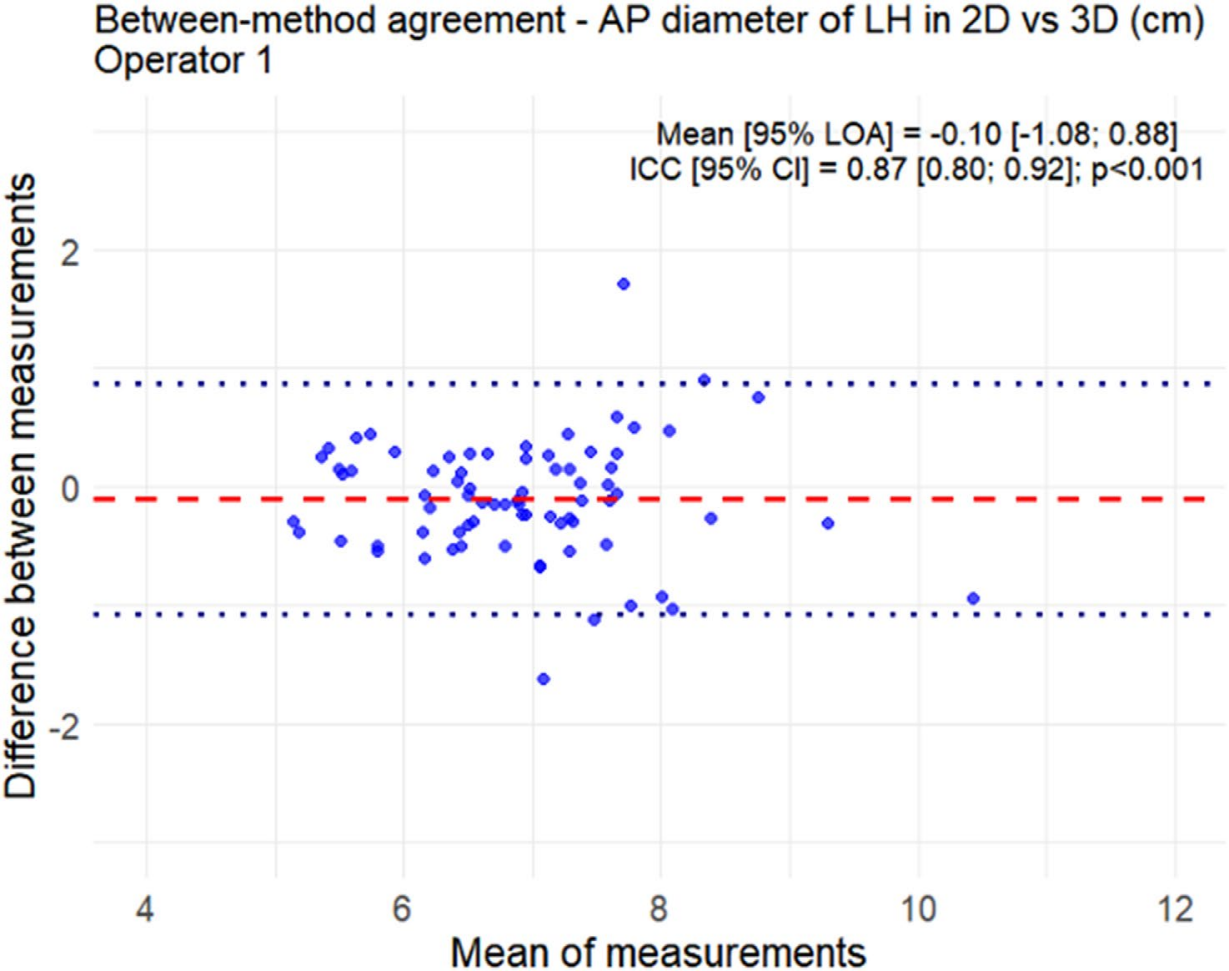
Bland–Altman plot with mean difference between the anteroposterior (AP) diameter of levator hiatus (LH) measured using 2D ultrasound compared with the three‐dimensional (3D) render for operator 1.

**FIGURE 7:**
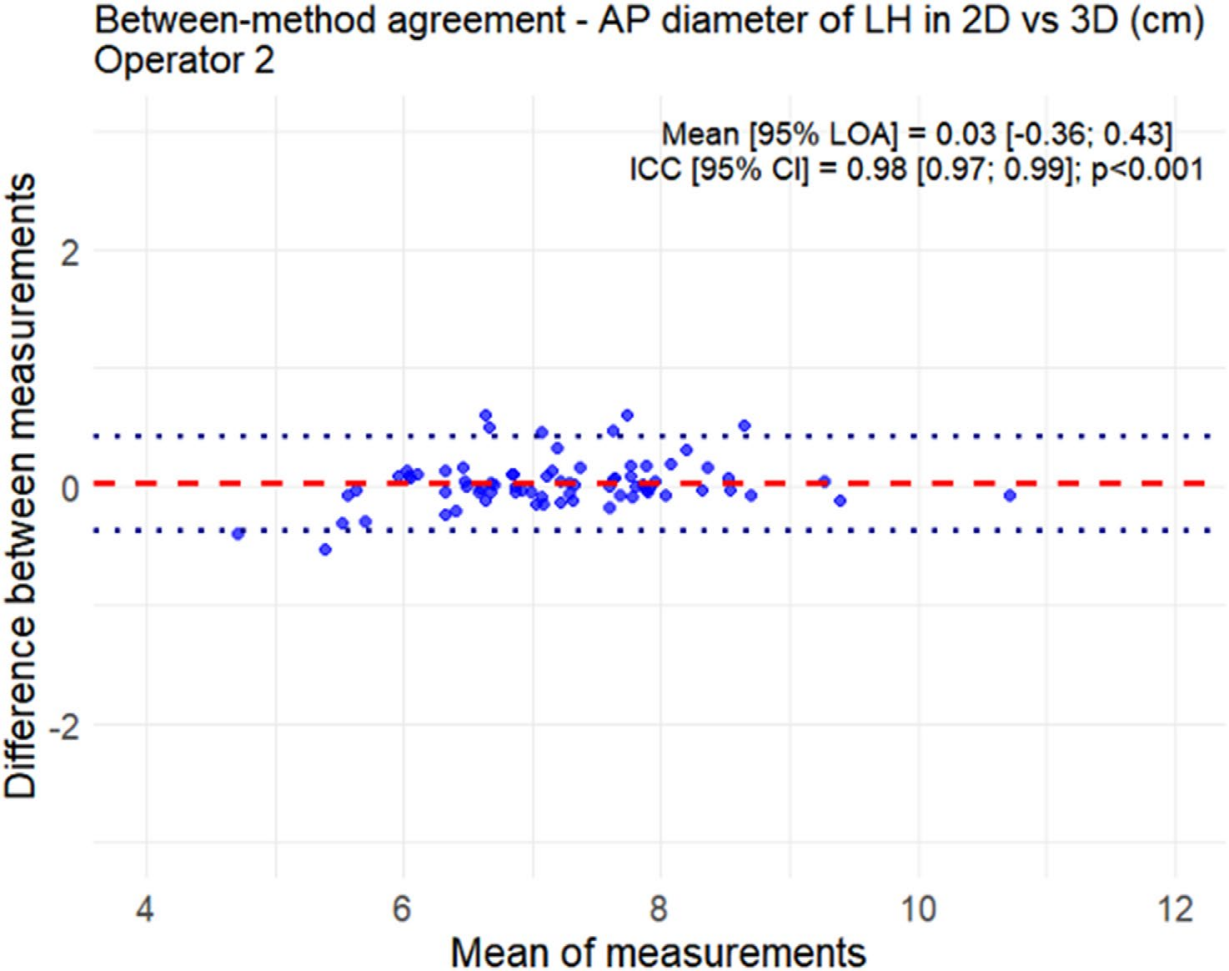
Bland–Altman plot with mean difference between the anteroposterior (AP) diameter of levator hiatus (LH) measured using 2D ultrasound compared with the three‐dimensional (3D) render for operator 2.

When comparing agreement between each operator, when operator 1 had undertaken the APD measurement, the difference observed between 2D and 3D measurements had a mean difference of −0.10 cm (ICC = 0.87; p < 0.001) and for operator 2 had an observed mean difference of 0.03 cm (ICC = 0.98; p < 0.001) (Figures 6 and 7).

### 3.2 Part 2: Quality assessment score for the anatomical structures identified from 3D hiatal volumes

The total score agreement between two observers was 72%, and was lower than individual scores for SP, PR, and LHa, which may reflect inter‐rater reliability across different measurements. The highest agreement was for SP at 88% (Kappa 0.4), followed by LHa at 86.7% (Kappa 0.77) and PR at 84.0% (Kappa 0.72). PR and LH show substantial agreement with the highest Kappa values (0.72 and 0.77). Despite SP having the highest observed agreement (88%), Kappa indicated only fair agreement (Kappa = 0.40): This combination is attributable to the prevalence effect, in which agreement is mainly driven by the dominant category rather than uniform classification across categories. All results are statistically significant (*p* < 0.001) (Table 3).

**TABLE 3:** Inter‐observer agreement between two independent practitioners of transperineal ultrasound, grading each three‐dimensional (3D) pelvic floor ultrasound render out of a total possible score of 8.

Score	Agreement (%)	Kappa	SE	*p* Value
SP	88.0%	0.40	0.12	< 0.001
PR	84.0%	0.72	0.09	< 0.001
LH	86.7%	0.77	0.09	< 0.001
Total	72.0%	0.61	0.07	< 0.001

Abbreviations: LH, Levator hiatus; PR, Puborectalis; SP, Symphysis pubis.

Although SP demonstrated the highest observed agreement (88%), the corresponding Kappa value indicated only fair agreement (Kappa = 0.40). This apparent discrepancy reflects a prevalence effect, whereby agreement is predominantly driven by the dominance of a single scoring category. Specifically, a score of 2 was assigned in the majority of cases by both observers (66/75 and 67/75, respectively). Thus, in this specific context, percentage agreement represents a more clinically informative measure of inter‐observer agreement than Kappa alone.

## 4 DISCUSSION

There is good inter‐ and intra‐observer agreement amongst all pelvic floor measurements.^22^ The inter‐observer agreement for APD in 3D had slightly better agreement compared with APD in 2D measurements. We hypothesize that the borders of the levator hiatal dimensions are more clearly visualized in 3D imaging due to the render more clearly defining the symphysis pubis and the puborectalis muscle. In comparison with 2D imaging, the clinician has to define the structures amongst gray‐scale imaging, which may be less clear. When comparing APD measurements between each operator between 2D and 3D imaging, good agreement was found; therefore, in practice, either technique is appropriate for obtaining the measurement, depending on the practitioner skillset.

Arguably, the most challenging measure of the levator hiatal dimensions is the LHa, which requires the operator to trace the levator hiatus in a point‐by‐point circumferential technique. This also has an observed good inter‐ and intra‐observer agreement.

In an Icelandic study by Eggebo et al.,^23^ the authors analyzed the levator hiatal TD from 78 women across all stages of labor. They did not report any significant correlation with the measurement and fetal occiput position, AoP, birthweight, or duration of the second stage of labor. This study proposed that the levator hiatal TD was consistent across certain labor variables. However, further work is needed to assess the rest of the pelvic floor dimensions (APD and LHa) with maternal and fetal characteristics. In a study by García Mejido et al.,^24^ twenty‐one participants were studied across labor stages, demonstrating that pelvic floor assessment is possible in the second stage of labor. However, the study numbers were small. This group suggested that there was an increase in the LHa as the fetal head station is lower.

The scoring methodology that we developed can standardize pelvic floor imaging not only in labor but outside the pregnancy setting. A clearly defined image specification is crucial to ensure that appropriate image capture has occurred before using the information to make clinical decisions. Additionally, a scoring system could be useful when teaching and training in 3D transperineal ultrasound of the pelvic floor to reinforce the landmarks which should be visualized. The total score that we have chosen as “acceptable” of ≥4, while arbitrary, is based on an analysis of the key landmarks required to perform hiatal measurements. Three components of anatomical structures should be visualized prior to undertaking levator hiatal measurements (symphysis pubis, puborectalis, and the outline of the levator hiatus). Through our analysis of several volumes, when the symphysis pubis and puborectalis outlines were only partially visible and/or obscured by shadowing, it was still possible to position the calipers for measuring. Therefore, we allocated this a score of 1 out of 2. In the outline of the levator hiatus, this structure was divided into four quadrants. Optimally, all four borders are visualized; however, as this is not always possible, if at least two borders are seen, the remaining two can be measured by approximation through awareness of the other landmarks. In the opinion of the authors, this constitutes the minimum requirement to obtain a measurement of the levator hiatal dimensions.

The strengths of this study include its prospective design in a cohort of women in the passive second stage of labor between uterine contractions. 3D volumes were examined with blinding between operators and the labor outcomes. Concurrent ultrasound and data collection were undertaken to obtain ultrasound findings of fetal head station and fetal head position. In future work, it would be interesting to evaluate the differences between maternal and fetal characteristics in relation to the pelvic floor measurements as this was not an objective of the current study.

Limitations are that we did not assess hiatal dimension changes with maternal pushing or post‐delivery imaging of the pelvic floor. In some cases, 3D volumes were excluded from analysis due to shadowing of the fetal head obscuring the image of the posterior portion of the levator hiatus. This was taken into consideration when developing the proposed scoring system, as the puborectalis muscle is an integral landmark to view when measuring APD and LHa. As such, the participants excluded from the study where the 3D volume was of insufficient quality may introduce a bias toward cases with better visualization.

## 5 CONCLUSION

Assessment of the levator hiatal dimensions in the second stage of labor is feasible with good inter‐ and intra‐observer agreement, and it supports the use of transperineal ultrasound in future studies of pelvic floor anatomy.

## AUTHOR CONTRIBUTIONS

**Mariya Kovalenko:** conceptualization, data curation, investigation, methodology, project administration, visualization, writing – original draft and review/editing. **Giulia Zamagni:** formal analysis, methodology, visualization, resources, writing – original draft and review/editing. **Ingrid Volløyhaug:** conceptualization, methodology, investigation, supervision, writing – review and editing. **Claudia Montero Carreras:** data curation, investigation, visualization, writing original draft and review/editing. **Sana Usman:** conceptualization, methodology, project administration, supervision, writing – review and editing. **Arwa Hanidu:** investigation, methodology, supervision, writing – review and editing. **Tamara Stampalija:** conceptualization, methodology, supervision, writing – review and editing. **Lorenzo Monasta:** formal analysis, visualization, methodology, writing – review and editing. **Kjell Salvesen:** conceptualization, methodology, supervision, writing – review and editing. **Christoph Lees:** conceptualization, methodology, project administration, resources, formal analysis, supervision, writing – review and editing.

## FUNDING INFORMATION

Christoph Lees is supported by the National Institute for Health Research (NIHR) Biomedical Research Centre based at Imperial College Healthcare NHS Trust and Imperial College London. The views expressed are those of the author (s) and not necessarily those of the NHS, the NIHR, or the Department of Health. Arwa Hanidu was supported by a grant funded by the Princess Nourah bint Abdulrahman University and Saudi Embassy (grant number: P90027).

## CONFLICT OF INTEREST STATEMENT

The authors declare no conflicts of interest.

## ETHICS STATEMENT

The study received UK Ethical approval from the Research Ethics Committee (REC) on November 14, 2022 (Reference 22/EM/0224) and Health Regulation Authority (HRA) approval on December 1, 2022 (Reference: 20/EM/0298). Local Joint Research Compliance Office approval was granted on January 22, 2023 (Reference: 20QC6287).

## Supporting information


**Figure S1.** Distribution of levator hiatal dimensions summarized by mean, standard deviation, median, interquartile range, and range (minimum–maximum).
**Figure S2**. Histograms showing the distribution of APD in 2D and 3D, LH area and TD.
**Figure S3**. Bland–Altman Plot with mean difference of the inter‐observer agreement in the anteroposterior diameter (APD) of the levator hiatus during three‐dimensional (3D) imaging.
**Figure S4**. Bland–Altman Plot with mean difference of the intra‐observer agreement in the anteroposterior diameter (APD) of the levator hiatus during three‐dimensional (3D) imaging.
**Figure S5**. Bland–Altman Plot with mean difference of the inter‐observer agreement in the levator hiatal (LH) transverse distance during three‐dimensional (3D) imaging.

## Data Availability

The data that support the findings of this study are available from the corresponding author upon reasonable request.
