# Supplementary material for: Inter‐ and intra‐observer agreement of 2D and 3D transperineal ultrasound of pelvic floor hiatal measurements in the second stage of labor
Source: Acta Obstet Gynecol Scand. 2026 Jun 16;105(7):1328–37. doi: 10.1111/aogs.70270 (PMC13308982; doi:10.1111/aogs.70270)
Supplement: Supplementary file 1 — Figure S1. Distribution of levator hiatal dimensions summarized by mean, standard deviation, median, interquartile range, and range (minimum–maximum). Figure S2. Histograms showing the distribution of APD in 2D and 3D, LH area and TD. Figure S3. Bland–Altman Plot with mean difference of the inter‐observer agreement in the anteroposterior diameter (APD) of the levator hiatus during three‐dimensional (3D) imaging. Figure S4. Bland–Altman Plot with mean difference of the intra‐observer agreement in the anteroposterior diameter (APD) of the levator hiatus during three‐dimensional (3D) imaging. Figure S5. Bland–Altman Plot with mean difference of the inter‐observer agreement in the levator hiatal (LH) transverse distance during three‐dimensional (3D) imaging. [file AOGS-105-1328-s001.docx]

**Supporting Information Legends**

| Variables | Mean | SD | Median | IQR | Min | Max |
| --- | --- | --- | --- | --- | --- | --- |
| APD (cm) in 2D | 7.05 | 1.05 | 7.15 | 1.41 | 4.63 | 10.35 |
| APD (cm) in 3D | 7.07 | 1.02 | 7.18 | 1.44 | 4.80 | 10.72 |
| LHa (cm^2^) in 3D | 20.40 | 6.00 | 19.55 | 6.92 | 9.61 | 50.11 |
| TD (cm) in 3D | 3.98 | 0.55 | 3.95 | 0.65 | 2.74 | 5.80 |

S1: Distribution of levator hiatal dimensions summarized by mean, standard deviation, median, interquartile range, and range (minimum–maximum).


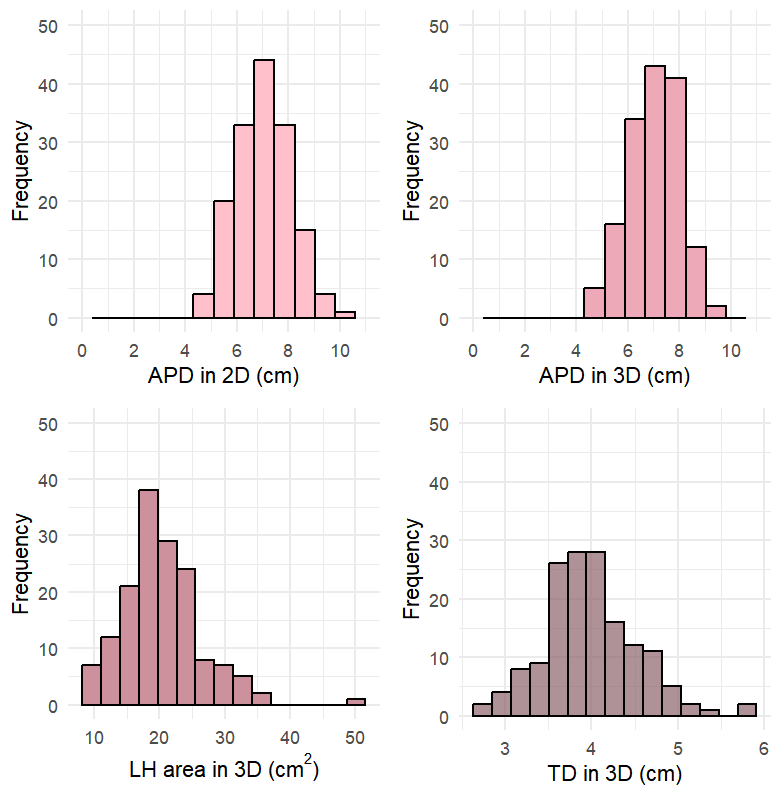


S2: Histograms showing the distribution of APD in 2D and 3D, LH area and TD.

 
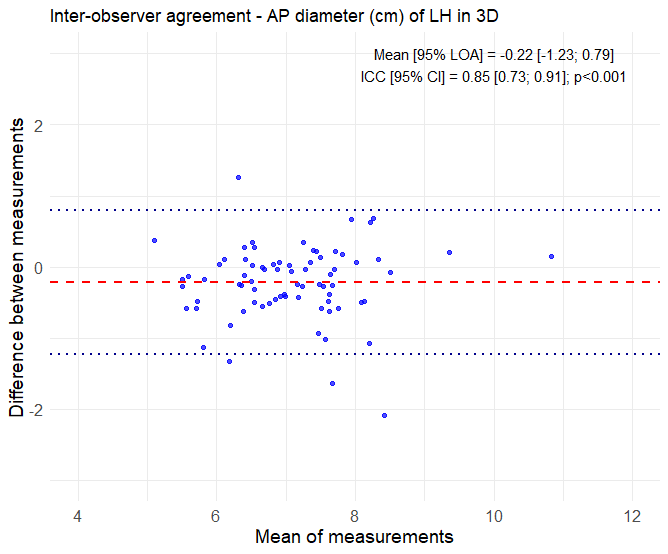


S3: Bland-Altman Plot with mean difference of the inter-observer agreement in the anterioposterior diameter (APD) of the levator hiatus during three dimensional (3D) imaging.


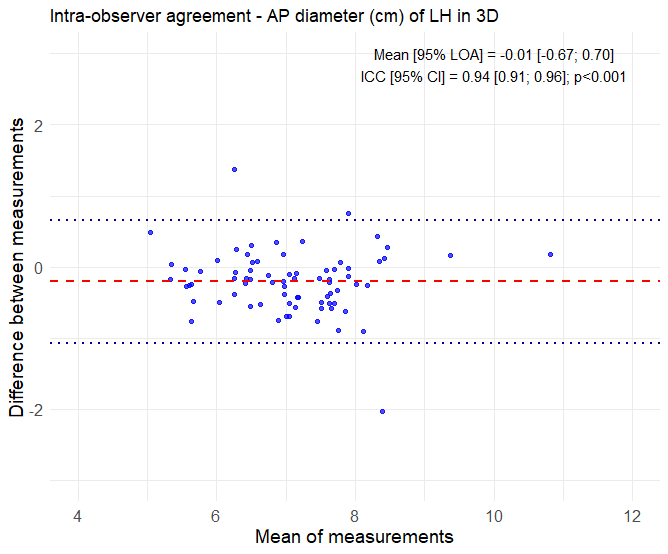


S4: Bland-Altman Plot with mean difference of the intraobserver agreement in the anteroposterior diameter (APD) of the levator hiatus during three dimensional (3D) imaging.


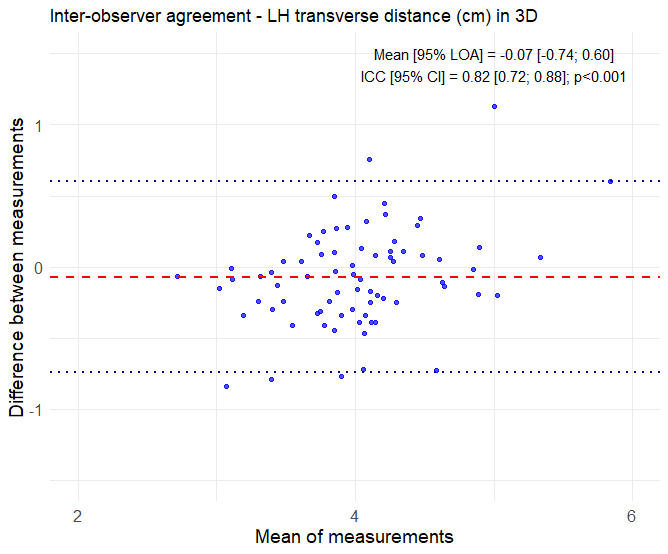


S5: Bland-Altman Plot with mean difference of the inter-observer agreement in the levator hiatal (LH) transverse distance during three-dimensional (3D) imaging.
